# Development of the Practice of Pharmaceutical Care for Cancer Pain Management in Outpatient Clinics Using the Delphi Method

**DOI:** 10.3389/fphar.2022.840560

**Published:** 2022-06-02

**Authors:** Lu Zhang, Xia-Yang Ren, Hang-Xing Huang, Ya-Min Huang, Ling Huang, Xiao-Ping Chen, Yao Chen, Chen Wang, Jian Xiao

**Affiliations:** ^1^ Department of Pharmacy, Xiangya Hospital, Central South University, Changsha, China; ^2^ Institute for Rational and Safe Medication Practices, National Clinical Research Center for Geriatric Disorders, Xiangya Hospital, Central South University, Changsha, China; ^3^ Department of Pharmacy, National Cancer Center/National Clinical Research Center for Cancer/Cancer Hospital, Chinese Academy of Medical Sciences and Peking Union Medical College, Beijing, China; ^4^ Department of Clinical Pharmacology, Xiangya Hospital, Central South University, Changsha, China; ^5^ Department of Pharmacy, Tianjin Medical University Cancer Institute and Hospital, Tianjin, China

**Keywords:** cancer pain, pharmaceutical services, ambulatory care, Delphi technique, care practice

## Abstract

**Background:** There exists no broad agreement of experts on the practice of pharmaceutical care for cancer pain management in outpatient clinics.

**Objectives:** This study aimed to use the Delphi consensus process to provide expert recommendations on the practice of cancer pain management in outpatient clinics from the point of view of pharmaceutical care in clinical practice and future clinical trials.

**Methods:** A comprehensive literature review was conducted to draft the initial practice. In this process, 30–40 senior experts from various provinces in China were invited to rank the items of practice during the two Delphi consultations. The definitions of consensus included a combination with an average score of ≥4, the percentage of experts rating the scores at >4 points, and the coefficient of variation of the scores.

**Results:** The expert panel comprised 18 pharmacists, 3 anesthesiologists, 6 oncologists, and 9 nurses. As a result of a comprehensive review, 33 items were initially formed. Among them, the consensus was reached for 27 items after the first Delphi round. The other six items and a total of five items for supplementation entered the second round, among which consensus was reached for eight items and three items were excluded. Expert consensus was achieved on 35 items after two rounds of consultation, which involved the collection of patient basic information, comprehensive pain assessment, breakthrough or neuropathic pain assessment, analgesic treatment evaluation, out-of-hospital follow-up, medical records, and evidence-based documents for reference.

**Conclusion:** The final list of 35 items could be used to develop the practice of pharmaceutical care for cancer pain management in outpatient clinics in China. The practice may aid in the standardization of pharmaceutical care for pain, relieve pain to the greatest extent possible, and enhance the level of pain management in China.

## 1 Introduction

With the increase in morbidity and mortality, cancer has become a major public health threat. In 2020, 19,292,789 new cancer cases are projected to occur worldwide (Xia et al., 2022). In China, there were approximately 4.57 million new cancer cases and 3 million cancer deaths (International Agency for Research on Cancer, 2020). Many patients with cancer experience pain during the development of the disease. The pain prevalence rates were 39.3% after curative treatment, 55.0% during anticancer treatment, and 66.4% in advanced, metastatic, or terminal disease (Kwon, 2014).

The three-step ladder for cancer pain treatment proposed by the World Health Organization (WHO) laid the foundation for standardized treatment. However, pain management in outpatient clinics, which is an important link between hospitalization and home care, continues to face multifactorial barriers. Medical staff is rarely skilled in comprehensive pain assessment and does not pay attention to the emotional and psychological status of the patients (Xia, 2017). The staff is inadequately educated and does not have sufficient knowledge of pain management. The patients have concerns about the adverse drug reactions (ADRs) and addiction (Lou and Shang, 2017). Most practice places are restricted to outpatient clinics, whereas some patients receive related care in the community to reduce general practice burdens (Allsop et al., 2018). Moreover, there are no universal medical documents, although they are important payment evidence that embody the value of the pharmacists, augment patient satisfaction, and promote the continuity of services (Houle et al., 2014).

The presence of clinical pharmacists in a multidisciplinary team (MDT) will ensure that patients receive standardized and individualized treatment plans. Studies have reported the positive impact of the participation of clinical pharmacists in reducing readmission rates, preventing adverse drug events, and alleviating medical care costs (Vulaj et al., 2018; Perrot et al., 2019). Compared with other medical staff, clinical pharmacists are more proficient in pain-related scales, and they are better at performing continuous assessments (Poirier et al., 2019). Their professionalism in the use of analgesic drugs and dosage adjustment enable them to recognize, solve, and prevent drug-related issues actively (Hadi et al., 2014). Coupled with the emphasis on medication education, the participation of clinical pharmacists in pharmaceutical monitoring will ensure the safety and rationality of drug use (Zhang et al., 2020).

Hepler and Strand defined pharmaceutical care as “the responsible provision of drug therapy for the purpose of achieving definite outcomes that improve a patient’s quality of life” (Hepler and Strand, 1990). The European Association of Pharmaceutical Care Network Europe (PCNE) defined it as “the contribution made by pharmacists to optimize the use of drugs and improve the health of patients” (Allemann et al., 2014). The core of pharmaceutical care is that pharmacists perform a pharmacological examination to identify, solve, and prevent drug-related problems (DRPs). The care aims to optimize medication use and improve health outcomes (Yap et al., 2009). The safety and effectiveness of the medications are ensured *via* closed-loop management that involves collecting patient information, identifying the present diagnostic and therapeutic problems, setting the treatment goals, identifying the DRPs, formulating/adjusting the treatment plans, implementing the plans, recording the monitoring behaviors, evaluating the curative effect and adverse reactions to determine whether the goals are achieved, and if not, redetermining drug-related issues (Medication therapy management in pharmacy, 2008). From the perspective of pain management, patient pain conditions are routinely evaluated; quantification scales are selected; the cause, type, location, quality, and aggravating or alleviating factors of pain are examined comprehensively; the changes in pain symptoms are assessed; and the presence of neuropathic or breakthrough cancer pain (BTcP) is identified (Swarm et al., 2019). Before formulating analgesic plan, drug therapy problems and ADRs should be checked (Scarborough and Smith, 2018). Furthermore, medication adherence should be evaluated to provide targeted education based on knowledge deficits to the patients and their caregivers (Oldenmenger et al., 2018). Finally, medical records should be created in a timely manner. Several studies have reported that pharmaceutical care exerts a positive impact on improving the patient pain control, preventing adverse drug events and reducing patient readmission rates and health care costs (Ni et al., 2018).

In China, more than 220 hospitals have established various pharmacy clinics as of 2019, and physician–pharmacist cancer pain management clinics for patients with cancer were one of the most typical clinics providing pharmaceutical care services (Hadi et al., 2014). However, almost every hospital has its practice model for pain management. In other countries, considering the gaps between the clinical and social environment, the differences in patient understanding of pain, the preferences for analgesic drugs, the subjective use of quantitative tools, and the models adopted by other countries are not well-applicable to the Chinese population (Grilli et al., 2000). The key issue is to reach a consensus to identify the best practice of pharmaceutical care for cancer pain management in outpatient clinics in China. Therefore, the objective of this study was to use a consensus process to provide expert recommendations on the practice of cancer pain management in outpatient clinics from the perspective of pharmaceutical care that can be applied in clinical practice and future clinical trials.

## 2 Methods

We conducted a comprehensive search of multiple literature databases to draft the initial practice of pharmaceutical care for cancer pain management in outpatient clinics. The Delphi method was adopted as it is a systematic approach to achieve a consensus among experts through independent completion of sequential questionnaires that were then refined based on the feedback, resulting in the convergence of opinions and eventual consensus. This study was approved by the Guidance on Conducting and Reporting Delphi Studies (CREDES). There was no requirement to acquire ethical approval for creating this consensus-based list (Wang et al., 2019).

Data collection was planned between March 2020 and April 2021. All collection rounds were completed electronically and anonymously with questionnaires and a secure web application for building and managing online surveys. The experts rated their agreement with statements in rounds 1 and 2 on a 5-point Likert scale.

### 2.1 Literature Review

The databases searched include PubMed, Web of Science, China National Knowledge Infrastructure (CNKI), and some guideline search websites (such as the National Guideline Clearinghouse (NGC), the Guidelines International Network (GIN), and the National Institute for Health and Care Excellence [NICE]) to identify the literature published before March 2020 in English or Chinese language. The following search terms were used: “cancer pain,” “pain management,” “pharmaceutical services,” “care practice,” “ambulatory care,” and “outpatient care.” Google Scholar allowed the inclusion of gray literature to identify some related content about the practice of pharmaceutical care for cancer pain management developed by different organizations (Shawahna, 2019; Shawahna, 2021). We excluded the publications that involved nonmalignant pain, did not mention any pharmaceutical care or medication, and did not describe any outpatient settings.

### 2.2 Expert Panel Selection

A judgmental sampling technique was utilized to identify, approach, invite, consent, and recruit experts to the panel. The design and aims of this study were explained to the candidate panelists by the field researchers (Shawahna et al., 2019). To avail expert opinion while drafting our final list and ensure that it is a useful and practical one, we invited experts from different geographical regions through an internet search. We aimed to invite 30–40 senior experts (Keeney et al., 2006), including clinical pharmacists, anesthesiologists, oncologists, and nurses from different provinces across China. All experts were required to meet the following inclusion criteria: 1) came from a tertiary hospital in China, 2) had at least 5 years of clinical experience of cancer pain, 3) held academic positions in provincial cancer pain–related associations, and 4) provided a guarantee to complete two rounds of questionnaires. To give play to the representativeness of pharmacists, the number of pharmacists recruited will be half of the total number of experts. To prevent overrepresentation from other expert groups, recruitment was monitored to achieve an approximate 50/50 split between physicians and nurses (Price et al., 2020). Invitations were sent to the experts *via* email.

### 2.3 The Initial Questionnaire

The initial questionnaire on the practice of cancer pain management in outpatient clinics was developed based on a literature review, and two pharmacists with background knowledge of cancer pain drafted it. They are specialized clinical pharmacists in the direction of cancer pain at the Xiangya Hospital, Central South University (an academic tertiary teaching hospital in the Chinese Hunan province), and one of them held academic background in oncology. The other three non-participants were required to unify language terms and test the time taken to complete the questionnaire.

### 2.4 Data Collection

During all survey rounds, the experts were asked to rate their opinion on a 5-point Likert scale (graded as strongly agree, agree, not necessarily, disagree, or strongly disagree). The definitions of consensus were a combination of an average score ≥4, the percentage of experts rating the scores at >4 points, and the coefficient of variation of the scores. The experts were also asked to self-rate themselves on the authority (Cr) for each round, which was determined by the judgment criteria (Ca) and their familiarities (Cs) with the clinical issues (Jiang et al., 2020). Ca includes four dimensions: work experience, theoretical analysis, understanding from domestic and foreign counterparts, and insights, and Cs include five levels: very familiar, familiar, generally familiar, unfamiliar, and very unfamiliar, which were quantified as 1.0, 0.8, 0.6, 0.4, and 0.2, respectively (Supplementary Table S1). The expert opinion coordination coefficient (W) was collected in these two rounds (Chen et al., 2016). The degree of positivity indicated the response rate, with >75% indicating meeting the standard value. The questionnaires were delivered to the experts individually *via* email. In order to ensure the response rate, each Delphi round was kept open for 2 weeks, with reminders being emailed at the beginning and end of every 2 weeks.

### 2.5 Delphi Rounds

#### 2.5.1 The First Round

An email containing the study details and a web link to an online questionnaire (using Select Survey. NET) were sent to the experts in March 2020. The instructions were provided on the first page of the questionnaire along with questions on the expert’s gender, age, education, occupation, job title, and years of work. The main body of the questionnaire consisted of a 5-point Likert scale. The initial practice of pharmaceutical care for cancer pain management in the outpatient clinics, which had 33 clinical themes, was ranked. The items described the method of collection of basic patient information (theme A), the method of assessing pain (theme B and C), the method to evaluate the analgesic treatment plan (theme D), the method to follow-up out-of-hospital (theme E), need of medical records (theme F), and the documents that could be used as evidence-based references (theme G). In addition, there was a space under each component for the experts to provide their comments, if any. After the completion of the first-round questionnaire, the average score and coefficient of variation were calculated. The definitions of consensus are as follows: 1) an average score ≥4.0, 2) at least 75% of the experts having rated “agree” or “strongly agree,” 3) coefficient of variation <0.15, and 4) no other objections. With at most 25% of the experts rated “agree” or “strongly agree,” the average score was <3.0; then, the items would enter in the second round. The experts were encouraged to raise more clinical issues of concern, and the results of the first round were fed back to each expert for reference during the second round. In the last part of the questionnaire, the experts were required to conduct a self-evaluation of their authority and the coordination coefficient.

#### 2.5.2 The Second Round

The items on which consensus was reached in the first round were not discussed any further, and all equivocal factors in the first round were considered in the second round (Cassar Flores et al., 2014). We reminded the experts of their scores and the numbers and percentages of the scores of other experts as well. The experts were asked whether they wished to reconsider their scores from the perspective of the scores and comments of other experts (Shawahna et al., 2020; Shawahna et al., 2021). In addition, the experts in the first round may propose to modify the expression of certain clinical questions or re-propose new clinical questions, which were combined to create a personalized second-round questionnaire. With the agreement of >75% of the experts, the average score was ≥4.0, and coefficient of variation was <0.15, and these items in the second-round questionnaire were included. The expert authority coefficient and opinion coordination coefficient were also calculated.

### 2.6 Statistical Analyses

Microsoft Excel 2016 and SPSS 23.0 were used to collect and analyze the data. Expert positive coefficient = (number of questionnaires returned/number of questionnaires sent) × 100%. Cr = (Ca + Cs)/2. The expert opinion coordination coefficient (W) was expressed by Kendall’s W, and the differences were compared using the chi-squared (χ^2^) test. *p* < 0.05 was considered to indicate statistical significance.

## 3 Results

### 3.1 Review of Literature: Establishment of an Initial Selection List

Our initial search identified a total of 5,816 related articles. After screening the titles and abstracts for studies that did not mention the practice of pharmaceutical care for cancer pain management in outpatient clinics, 312 articles were finally included. After studying the full text of the articles, we formed an initial practice list including 33 items.

### 3.2 Basic Characteristics of the Experts

As presented in Table 1, 36 experts, including 18 pharmacists, 3 anesthesiologists, 6 oncologists, and 9 nurses, were invited to this study. The experts hailed from tertiary hospitals in nine provinces in China. The male to female ratio was 1:5. The experts over 40 years of age accounted for 72.2% (26/36). Their average experience in the field of cancer pain was 12 years, and 63.9% of the experts had an experience of >10 years. All experts had received higher education and worked as associate directors or in a higher position.

**TABLE 1:** Baseline characteristics of the experts.

	N	%
**Profession**		
Pharmacist	18	50.0
Physician (anesthesiologists)	3	8.3
Physician (oncologists)	6	16.7
Nurse	9	25.0
**Age, y**		
30-39	10	27.8
40–49	20	55.5
50–59	6	16.7
**Gender**		
Male	6	16.7
Female	30	83.3
**Highest level of education**		
Bachelor’s degree	12	33.3
Master’s degree	13	36.1
Post-master’s degree (PhD)	11	30.6
**Professional title**		
Director	9	25.0
Associate director	27	75.0
**Work experience in cancer pain, y**		
5-9	13	36.1
10–19	18	50.0
20–29	3	8.3
≥30	2	5.6

### 3.3 Results of the Delphi Rounds

#### 3.3.1 The First Round

During the first Delphi round, the experts were invited to furnish their opinions on the 33 items included in the initial list (Table 2). All items were rated as “agree” or “strongly agree,” with an average score of ≥4.0. These items were grouped under seven categories. Of the items presented to the panelists in the first Delphi round, the consensus was achieved to include 27 (81.8%) items. Of these, three (11.1%) items were related to the basic information collection of the patient, nine (33.3%) items were related to comprehensive pain assessment, two (7.4%) items were related to further refractory pain assessment, three (11.1%) items were related to analgesic treatment evaluation, three (11.1%) items were related to the out-of-hospital follow-up, two (7.4%) items were related to medical records, and five (18.5%) items were related to evidence-based reference documents.

**TABLE 2:** Clinical questions of the first round.

No	Clinical question		Average score	Score>4 (%)	Coefficient of variation (%)	Reach a consensus
Theme A: Collection of patient basic information						
A1	Collect patient general information, clinical diagnosis, and auxiliary examination		4.39	91.8	0.22	NO
A2	Collect personal and allergic history		4.61	97.3	0.12	YES
A3	Collect tumor-related treatment		4.58	97.3	0.12	YES
A4	Collect performance status		4.61	97.3	0.12	YES
Theme B: Comprehensive pain assessment						
B1	Assess pain cause, location, intensity, and quality of pain		4.80	100	0.08	YES
B1-1		Pain cause, assess tumor- caused, tumor treat–caused, and non-tumor–related pain	4.72	97.3	0.11	YES
B1-2		Pain location, mark in the table directly	4.75	100	0.09	YES
B1-3		Pain intensity, assessed by quantitative tools	4.72	94.5	0.12	YES
B1-4		Pain quality, mark in the table directly	4.72	100	0.10	YES
B2	Pain worsening or alleviating factors		4.61	97.3	0.12	YES
B2-1		Worsening factors, including activities, weather, and mental factors	4.45	88.9	0.16	NO
B2-2		Alleviating factors, including takin g analgesics, suitable environment, and positive psychology	4.64	100	0.11	YES
B3	Differentiation of persistent pain		4.58	91.7	0.14	YES
B3-1		Assess onset time, interval, duration, and intensity of pain	4.64	94.5	0.13	YES
Theme C: Further refractory pain assessment						
C1	Neuropathic pain assessment					
C1-1		Assess quality of pain; hyperalgesia	4.58	97.3	0.12	YES
C2	BTcP assessment					
C2-1		Assess frequency, duration, and intensity and type of pain	4.69	100	0.10	YES
Theme D: Analgesic treatment evaluation						
D1	Assess drug indications and effectiveness		4.64	97.3	0.12	YES
D2	Assess adverse drug reaction		4.70	100	0.10	YES
D3	Assess medication adherence		4.61	97.3	0.12	YES
Theme E: Out-of-hospital follow up						
E1	Assess the 24-h use of analgesics		4.58	100	0.11	YES
E2	Assess the 24-h BTcP		4.47	94.5	0.14	YES
E3	Record physician or pharmacist recommendations		4.47	94.5	0.14	YES
Theme F: Medical records						
F1	Patient basic information form		4.53	94.6	0.19	NO
F2	Comprehensive pain, neuropathic pain, and BTcP assessment form		4.47	91.7	0.15	YES
F3	Drug therapy evaluation form		4.58	97.3	0.12	YES
F4	Out-of-hospital follow-up form		4.11	83.5	0.24	NO
Theme G: Evidence-based documents for References						
G1	Drug instructions		4.50	91.7	0.15	YES
G2	NCCN Guidelines Version 1.2020 Adult Cancer Pain		4.5	94.5	0.14	YES
G3	Management of cancer pain in adult patients: ESMO Clinical Practice Guidelines		4.36	86.2	0.18	NO
G4	WHO three-step analgesia guidelines		4.45	91.7	0.15	YES
G5	Cancer pain diagnosis and treatment specifications (Chinese)		4.58	94.5	0.13	YES
G6	Expert consensus on refractory cancer pain (Chinese)		4.47	94.5	0.14	YES
G7	UpToDate (CDSS)		4.22	80.6	0.18	NO

BTcP, breakthrough cancer pain; NCCN, National Comprehensive Cancer Network.; ESMO, European Society for Medical Oncology; CDSS, clinical decision support system.

The coefficient of variation was not reached for one item in theme A (basic information collection), one item in theme B (comprehensive pain assessment), two items in theme F (medical records), and two items in theme G (evidence-based reference documents). Hence, these items would enter in the second round. In addition, a total of five items were submitted by the experts for supplementation: 1) lifestyle of the patients, 2) contact details of the patients, 3) personal willingness, 4) drug accessibility, and 5) satisfaction survey, which were also included in the second round.

#### 3.3.2 The Second Round

Based on the results of the first-round rating, the Delphi consultation questionnaire was revised. The new questionnaire included 11 items (Table 3). Of the items presented to the panelists in the second Delphi round, a consensus was achieved to include eight (72.7%) items. These items were grouped under five categories. Of these, one (12.5%) item was related to the basic information collection of the patient, one (12.5%) item was related to comprehensive pain assessment, two (25.0%) items were related to the out-of-hospital follow-up, one (12.5%) item was related to evidence-based reference documents, and three (37.5%) items were related to others. In this round, a consensus was not reached on as the remaining question “UpToDate (clinical decision support system) could be used as an evidence-based reference for the practice of pharmaceutical care.” The newly raised questions on drug accessibility and personal willingness were also excluded because of the coefficient of variation.

**TABLE 3:** Clinical questions of the second round.

No	Clinical question	Average score	Score>4 (%)	Coefficient of variation (%)	Reach a consensus
Theme A1	Collect general information, clinical diagnosis, and auxiliary examination	4.69	100	0.10	YES
Theme B2-2	Worsening factors including activities, weather, and mental factors	4.78	100	0.09	YES
Theme F1	Form of patient basic information	4.53	94.5	0.14	YES
Theme F4	Form of out-of-hospital follow up	4.47	94.5	0.14	YES
Theme G3	Management of cancer pain in adult patients: ESMO Clinical Practice Guidelines	4.64	97.3	0.12	YES
Theme G7	UpToDate (CDSS)	4.03	80.7	0.20	NO
Other	Lifestyle of patients	4.47	94.5	0.14	YES
	Contact details	4.72	97.3	0.13	YES
	Personal willingness	4.36	91.8	0.24	NO
	Drug accessibility	4.33	89.0	0.23	NO
	Satisfaction survey	4.81	100	0.08	YES

BTcP, breakthrough cancer pain; NCCN, National Comprehensive Cancer Network; ESMO, European Society for Medical Oncology; CDSS, clinical decision support system.

#### 3.3.3 Delphi Consultation Results

**FIGURE 1:**
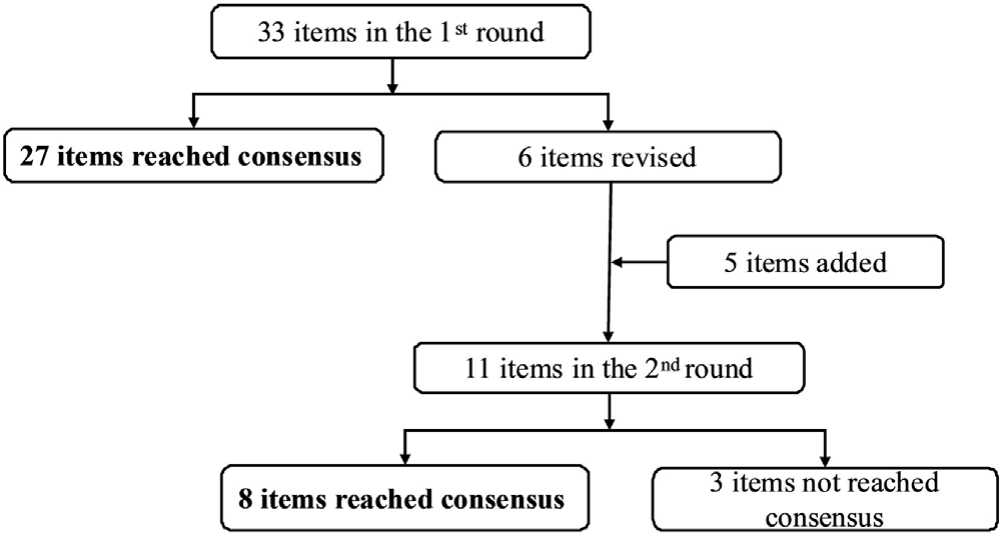
Overview of the Delphi rounds.

**FIGURE 2:**
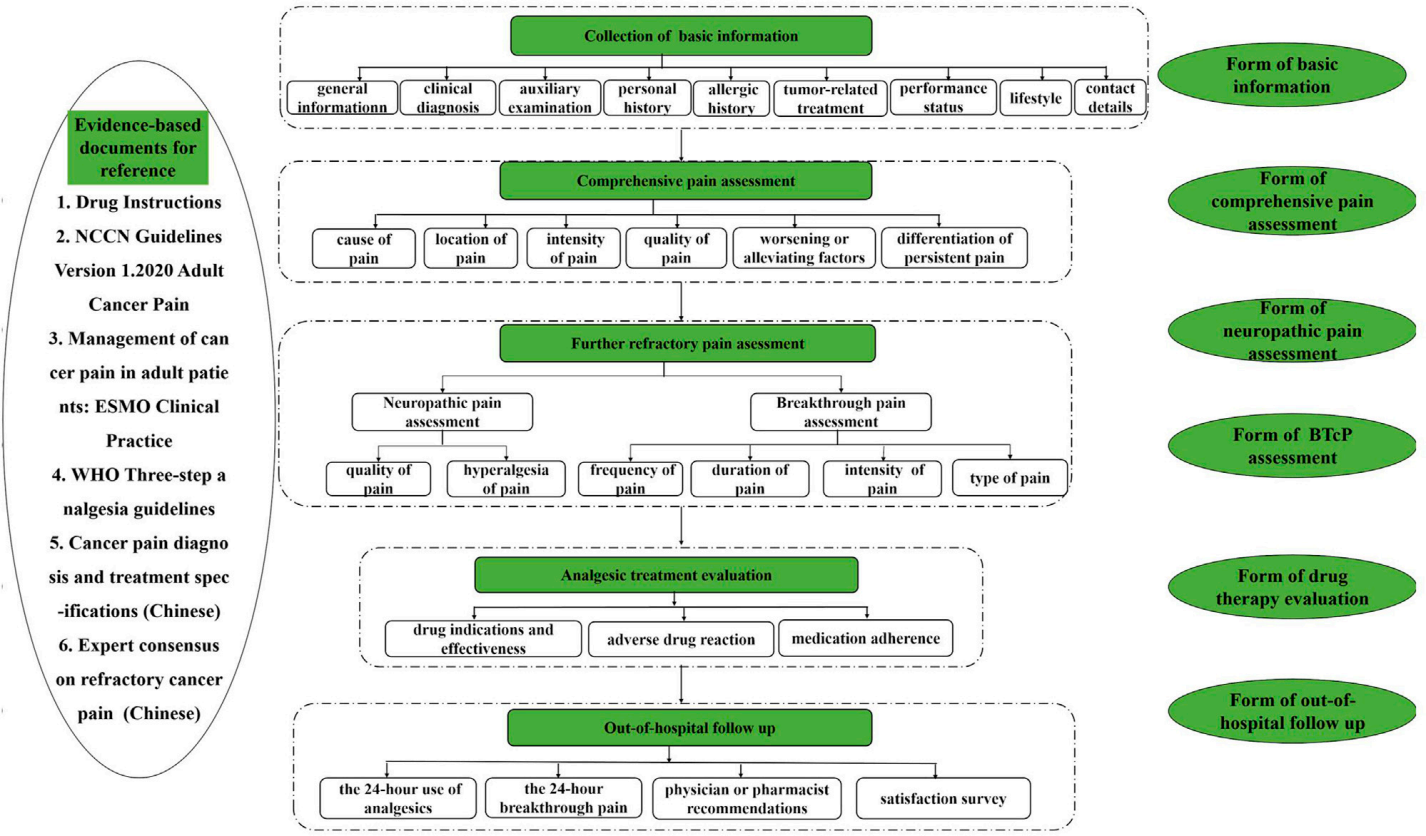
The flowchart of the practice of pharmaceutical care for cancer pain management in outpatient clinics.

The results of the rounds are summarized in Figure 1. The flowchart is depicted in Figure 2. After a comprehensive review, 33 items for the practice of pharmaceutical care for cancer pain management in outpatient clinics were initially formed. Among them, a consensus was reached after the first Delphi round for 27 items. The other six items and a total of five items submitted by experts for supplementation entered the second round. In the second round, a consensus was reached on eight items and three items were excluded.

After the two Delphi rounds, a consensus was achieved on 35 items for the practice of pharmaceutical care for cancer pain management in outpatient clinics, which integrated the relevant aspects of the collection of patient basic information, comprehensive pain assessment, BTcP or neuropathic pain assessment, analgesic treatment evaluation, out-of-hospital follow-up, medical records, and evidence-based documents for reference.

The response rate for both rounds was 100% (36/36). In the two Delphi rounds, the average value of expert familiarity (Cs) was >0.70 and the average value of the expert judgment criteria (Ca) and the authority coefficient (Cr) were both >0.80 (Tables 4, 5). The expert opinion coordination coefficient (W) was 0.098 in the first round and 0.103 in the second round. The χ^2^ test showed that the expert opinion coordination was significant (*p* < 0.001), implying that the two rounds of expert opinions were well-coordinated and the results were reliable (Table 6).

**TABLE 4:** Expert authority coefficient (Cr) in the first round.

Themes	Cs	Ca	Cr
Collection of basic patient information	0.70	0.88	0.78
Comprehensive pain assessment	0.80	0.88	0.83
Further refractory pain assessment	0.75	0.87	0.80
Analgesic treatment evaluation	0.70	0.88	0.83
Out-of-hospital follow up	0.80	0.85	0.76
Medical records	0.70	0.86	0.78
Evidence-based documents for References	0.80	0.88	0.83
Average value	0.75	0.87	0.80

Cs, the expert familiarity; Ca, the expert judgment criteria; Cr, the expert authority coefficient.

**TABLE 5:** Expert authority coefficient (Cr) in the second round.

Themes	Cs	Ca	Cr
Collection of basic patient information	0.80	0.86	0.84
Comprehensive pain assessment	0.80	0.85	0.83
Medical records	0.80	0.83	0.83
Evidence-based documents for References	0.80	0.87	0.84
Average value	0.80	0.85	0.84

Cs, the expert familiarity; Ca, the expert judgment criteria; Cr, the expert authority coefficient.

**TABLE 6:** Coefficient of concordance (W) of experts in each round.

Delphi round	Items	w	χ^2^	*p*
Round 1	33	0.098	30.905	<0.001
Round 2	11	0.103	115.536	<0.001

W, the coefficient of concordance.

## 4 Discussion

### 4.1 The Practice

In this study, a consensus was reached on the process/each step of provision of pharmaceutical care for cancer pain management. The practice of pharmaceutical care for cancer pain management in outpatient clinics was developed to bridge the literature gap in this field. Medical staff is expected to consider such consensus items while providing pharmaceutical care (Ahmedzai et al., 2019). In this study, a consensus was reached on the following items: the medical staff should evaluate patient basic information, assess pain comprehensively and identify whether there is neuropathic or breakthrough cancer pain, check drug problems before formulating an analgesic plan, and then conduct out-of-hospital follow up. Evidence-based documents should be used for reference, and medical records should be prepared for the entire process. Such information could be used to answer what and how questions, such as how to assess cancer pain and how to evaluate the analgesic treatment plan, and what medical records are needed.

To the best of our knowledge, detailed recommendations are presently not available on which items are important in the practice of pharmaceutical care for cancer pain management in outpatient clinics. Liu et al. formulated a pharmaceutical intervention workflow for cancer pain management, which divided the practice into four stages: inspection, diagnosis, treatment, and follow-up. However, as a typical attempt, the workflow did not include the refractory cancer pain assessment and relevant scales. Owing to the gaps in the clinical and social environment, the practices of other nations do not apply to the Chinese population (Grilli et al., 2000). Another study reached a consensus on pain assessment and management, but it did not consider the entire process of pharmaceutical care. Furthermore, it did not include the considerations of medical documents and evidence-based references (Varrassi et al., 2020). However, this study paid attention to the process of pain assessment and management in the real outpatient environment, which may have more potential in developing the standardized pharmaceutical care for pain and enhancing the level of pain management.

This study reached a consensus on the assessment of BTcP or neuropathic pain. Many studies had focused on the definitions, diagnosis, treatment, and the associated management of two specific types of pain. Even today, there is no validated method for assessment. This study proposed that the frequency and duration of episodes and monitoring of the intensity of pain should be included, which is in line with the literature reports (Boceta et al., 2016). However, the specific frequency of assessments, therapeutic options to be preferred for particular pain statuses, or preferred tools to be used should have been provided to enhance the impact of the study findings. The assessment varies from one clinical setting to the other based on the local experience. It is not possible to provide a detailed assessment of both types of pain with a unified procedure.

One study proposed that the best tool for assessing and monitoring pain is either a standardized document or some generic recommendations on patient outcome records for referral to the doctor (Boceta et al., 2016). So far, there are no universal medical documents in China, and this study provided a template to homogenize and support pain improvement. On the one hand, the completion of medical records by pharmacists or other medical staff was part of the standard practice. On the other hand, medical documents were important payment evidence that reflected the service value of the pharmacists. The medical documents ensured that all patients received appropriate patient-centered pharmaceutical care instead of stacking all items on a single form for the sake of seeking completeness.

Even though the latest WHO guidelines for cancer pain management called for attention to drug accessibility, a consensus was not reached on this item. The participants in another study seemed to support the notion that familiarity, availability, and cost should also be considered during the selection process of medications, but, similarly, a consensus was not reached (Varrassi et al., 2021). In our study, a consensus was not reached on the item of personal willingness either as it was related not only to the incomes of people in different regions of China but also to the attitudes toward the use of opioids (Muckenhuber et al., 2014). An example of patient concern was physical dependence. Another study raised a different view that the problem in treating cancer pain was making the appropriate choice and the use of these therapies, which meant that the supporting clinicians should deliver personalized treatments tailored to individual needs (Hui and Bruera, 2014).

### 4.2 Delphi Process

The Delphi method is useful in developing clinical questions of medical quality. There is no guidance that exists on the minimum or maximum number of experts on a panel and no formula to help us decide on how many there should be. Rather, as in other types of surveys that use nonprobability sampling techniques, the number of experts is often based on rigorous inclusion and exclusion criteria (Shawahna, 2020). The literature shows that too few experts lead to bias and too many experts introduce difficulties in data analysis. Hence, it is more appropriate to select 10–40 experts (Keeney et al., 2006). Wang et al. invited 36 experts, including 6 geriatricians, 6 anesthesiologists, 6 surgeons, and 18 pharmacists, to prepare a high-risk perioperative medication list (Wang et al., 2019). The percentage of physicians in another study who developed a trigger tool was 72.3% (13/18). We invited 36 experienced experts, all of whom had senior titles or above. Moreover, 63.9% (23/31) of the experts had worked on cancer pain for >10 years, which ensured the scientific nature of the research. Considering the role of pharmacists in the MDT and professionalism in pain management, 50% (18/36) of the experts in the group were pharmacists.

One study on developing a quality instrument for assessing the spontaneous reports of ADR/ADE had the following definitions of disagreement: indicators whose mean score was <3.0 and had a coefficient of variation of >0.25 were rejected (Chen et al., 2016). Clinical medication guidelines for high-dose methotrexate in China set the definitions of consensus in the first Delphi round as “mean score was ≥4, and the coefficient of variation was ≤0.15.” In another study, the items were retained if at least 80% of the respondents in all groups considered them important or if 90% consensus was reached in at least one group (Kinnaer et al., 2019). Considering the settings for the earlier study, the definitions of consensus for this study were a combination of an average score of ≥4, the percentage of experts rating the scores at >4 points, and the coefficient of variation of the scores.

The results showed that the response rate for both rounds was 100%, which indicated that the experts were interested in this research and were willing to complete the questionnaires within the limited time. An authority coefficient of >0.70 is generally considered to be acceptable (Wang et al., 2019). The coefficients of the expert authority for the first and second rounds in this study were 0.80 and 0.84, respectively, and the Cs was 0.75 and 0.80, respectively, which indicated that the experts were highly familiar with the questions. Previous studies have shown that the coordination coefficient (W) ranges from 0 to 1 (Wang et al., 2019). Our study demonstrated the values of 0.098 and 0.103 for the first and second rounds, respectively, which were statistically significant (*p <* 0.001). This result implied that consensus had been reached and that its strength was increasing and the determination of the relative strength of the consensus.

### 4.3 Limitations of the Study

This study has the following limitations: 1) the practice was based on literature research and the Delphi method. Hence, it could be subjective. 2) In the Delphi method, the number and representativeness of the experts are the important factors affecting the generalizability of the results. The panel comprised experts only from nine provinces in China. Thus, the universality of the results would be limited. Considering that the physicians included anesthesiologists and oncologists, the number of physicians among all the experts should be balanced. 3) Some themes in this study included more than one statement. In this case, expert agreement or disagreement may hold good for one of the statements and not for the theme itself. 4) The flowchart was not exhaustive for the treatment of cancer pain, including pharmacological and nonpharmacological interventions. 5) As this study was focused on the practice of pharmaceutical care for cancer pain management in outpatient clinics in China, the diagnosis of diseases and the issuance of prescriptions were not considered. 6) The practice has not yet been applied in a real clinical setting; hence, its role remains unclear.

### 4.4 Further Work

The practice developed in this study can be used to build clinical pathways or enhance the care processes. The different roles of the team members should be further described as also the communication, coordination mechanisms, and processes. Moreover, the care pathway should be promoted with the continuous quality and efficiency improvement processes occurring within the MDT.

The standardization of pharmaceutical care practice for cancer pain is only the first step. To truly improve adherence to the medication, we should embrace artificial intelligence and embed it in the actual clinical environment of physicians or pharmacists. A clinical decision support system (CDSS) is an interactive expert system based on the clinical knowledge and patient condition, which uses computer technology to provide warnings, reminders, and auxiliary diagnoses to assist medical staff in making medical decisions (Beeler et al., 2014). Like a pyramid of the level of evidence utilization, the CDSS has the potential to improve adherence to the practice and guide further improvements.

## 5 Conclusion

In this study, by combining literature with two rounds of consultations, expert consensus was achieved on the practice of pharmaceutical care for cancer pain management in outpatient clinics in China, which included 35 items related to the collection of patient basic information, comprehensive pain assessment, BTcP or neuropathic pain assessment, analgesic treatment evaluation, out-of-hospital follow-up, medical records, and evidence-based documents for reference. The practice may have the potential to develop standardized pharmaceutical care for pain, relieve pain to the greatest extent, improve patient quality of life, and enhance the level of pain management in China. In the future, information-based methods can be applied to better implement the pathway of pharmaceutical care, disseminate it to more regions and hospitals, and benefit more patients.

## Data Availability

The original contributions presented in the study are included in the article/Supplementary Material, further inquiries can be directed to the corresponding authors.
