# Supplementary material for: Development of the Practice of Pharmaceutical Care for Cancer Pain Management in Outpatient Clinics Using the Delphi Method
Source: Front Pharmacol. 2022 Jun 2;13:840560. doi: 10.3389/fphar.2022.840560 (PMC9201566; doi:10.3389/fphar.2022.840560)
Supplement: Supplementary file 1 [file Table1.docx]

Supplementary Table 1. Judgment criteria and degree of influence.

| **Judgment criteria** | **Degree of influence** | | |
| --- | --- | --- | --- |
|  | **High** | **Middle** | **Small** |
| Work experience | 0.5 | 0.4 | 0.3 |
| Theoretical analysis | 0.3 | 0.2 | 0.1 |
| Understanding from domestic and foreign counterparts | 0.1 | 0.1 | 0.1 |
| Insight | 0.1 | 0.1 | 0.1 |
